# Seroprevalence of hepatitis B and C viral co-infections among children infected with human immunodeficiency virus attending the paediatric HIV care and treatment center at Muhimbili National Hospital in Dar-es-Salaam, Tanzania

**DOI:** 10.1186/1471-2458-7-338

**Published:** 2007-11-22

**Authors:** Safila P Telatela, Mecky I Matee, Emmanuel K Munubhi

**Affiliations:** 1 Department of Paediatrics and Child Health, Muhimbili University College of Health Sciences, P.O. Box 65001, Dar es Salaam, Tanzania; 2 Department of Microbiology and Immunology, Muhimbili University College of Health Sciences, P.O. Box 65347, Dar es Salaam, Tanzania

## Abstract

**Background:**

With increased availability of antibiotics and antifungal agents hepatitis B virus (HBV) and hepatitis C virus (HCV) infections are becoming a cause for significant concern in HIV infected children. We determined the seroprevalence and risk factors for HBV and HCV among HIV infected children aged 18 months to 17 years, attending the Paediatric HIV Care and Treatment Center (CTC) at Muhimbili National Hospital (MNH) in Dar-es-Salaam, Tanzania.

**Methods:**

Investigations included; interviews, physical examination and serology for HBsAg, IgG antibodies to HCV and alanine aminotransferase (ALT) levels. HIV serostatus and CD4 counts were obtained from patient records.

**Results:**

167 HIV infected children, 88(52.7%) males and 79(47.3%) females were enrolled. The overall prevalence of hepatitis co-infection was 15%, with the seroprevalence of HBV and HCV being 1.2% and 13.8%, respectively. Hepatitis virus co-infection was not associated with any of the investigated risk factors and there was no association between HBV and HCV. Elevated ALT was associated with hepatitis viral co-infection but not with ART usage or immune status.

**Conclusion:**

The high seroprevalence (15%) of hepatitis co-infection in HIV infected children attending the Paediatrics HIV CTC at the MNH calls for routine screening of hepatitis viral co-infection and modification in the management of HIV infected children.

## Background

With more than 1.4 million women estimated to give birth annually and 8.2% HIV prevalence rate at antenatal clinics (ANC), approximately 122,000 HIV-infected women deliver annually in Tanzania. Assuming a 40% transmission rate in the absence of any intervention, an estimated 48,000 children will become HIV-infected each year. In 2003, the number of children (0–15 years) living with HIV/AIDS in Tanzania was estimated to be between 85,000 and 230,000[1,2]. A large number of infected children have been associated with significant increase in morbidity, hospitalization and mortality [2].

With increased access to antibiotics and antifungal agents hepatitis viruses, especially hepatitis B and C, are emerging as the leading causes of morbidity and mortality among children on ART [3].

Relatively little is known regarding HCV or HBV co-infection in HIV infected children in Tanzania. Thus, not surprisingly the current national guidelines for management of HIV disease in children do not include screening and management of hepatitis viral co-infection.

We conducted this study to determine the seroprevalence and risk factors for HBV and HCV among HIV infected children aged 18 months to 17 years attending the paediatric HIV Care and Treatment Center (CTC) at MNH, Dar-es-Salaam, Tanzania.

## Methods

### Study design, setting and population

This cross sectional study was conducted at the Paediatrics HIV CTC in Muhimbili National Hospital (MNH) between April 2006 and August 2006. The MNH is the largest referral and consultant hospital in Tanzania. All HIV infected children who were attending the Paediatrics HIV CTC at MNH during the study period were eligible. Children included in this study were aged between 18 months and 17 years.

### Interviews

Parents/guardians were interviewed using a standard structure questionnaire to obtain information regarding demographics, age, sex, and duration of illness, past medical history such as history of blood transfusion, and parenteral treatment, family and social history. Patient record files were used to obtain information regarding HIV status and CD4 count. Only recent CD4 values (within three months) were recorded. Children were classified immunologically according to the immunological classification for HIV-infected infants and children by United States Centers for Disease Control and prevention [4].

### Physical examination

A thorough physical examination was done according to standard clinical methods [5]. General examination, followed by systemic examination was done. General examination included looking at the skin and mucous membranes for the presence of jaundice especially the sclera and mucous under the tongue, palmar pallor, lymph node enlargement by palpation. Abdominal examination to elicit enlargement of the liver and spleen was done.

### Laboratory investigations

#### Detection of HBsAg and IgG antibody against hepatitis C virus

Detection of HBsAg was done using ELISA technique (EIAgen HBsAg Kit). IgG antibodies to HCV were detected using an ELISA technique (EIAgen HCV Ab Kit). The tests which were done according to instructions of the manufacturer (adaltis Italia S.p.A) have specificity and sensitivity of 99% and 100% respectively.

#### Alanine aminotransferase (ALT) assay

Catalytic activity of ALT (EC 2.6.1.2) was determined in serum using a COBAS MIRA chemistry analyzer (GMI, MI, USA) after it was calibrated.

### Statistical analysis

Data were entered, cleaned, and analyzed using EPI INFO version 3.3.2 and SPSS version 10.0 [6]. The seroprevalence of HCV and HBsAg were expressed in percentages for the entire study group and by age and sex. Chi-Square (χ^2^) was used to determine the association between level of immunosuppression with HBV and HCV. Fisher's exact test was used where the expected frequency was less than five. Univariate and multivariate regression were used to determine the associations between elevation of ALT, immunosuppression, ART and hepatitis co-infection. The associations were presented as odds ratio (OR) together with 95% confidence intervals (C1) and were considered to be significant if the corresponding 95% C1 does not include one.

### Ethical issues

An oral informed consent was obtained from the parent/guardian prior to enrollment and assent to those children more than 10 years was also obtained. The following information was given during parent/guardian education to ensure that they have the information needed to make an informed choice; a complete description of the aims of the study, infectious agents that were screened, potential benefits and risks, blood collection procedures and assurance of confidentiality of any information given as well as test results. Study personnel provided any other requested additional information to parent/guardian. Children with hepatitis co-infection were followed up at the clinic and cared and managed accordingly.

## Results

A total of 167 children were recruited between April and august 2005. Among them, 88(52.7%) were males and 79(47.3%) were females. Most of the children (49.7%) were aged 6–10 years. The overall prevalence of hepatitis viral co-infection among the HIV infected children was about 15% (25/167). The prevalence of hepatitis viral co-infection was significantly higher among girls (21.5%) than boys (9.0%). There was no association between age and hepatitis viral co-infection. Two children (1.2%) were co-infected with HBsAg and 23(13.8%) were co-infected with HCV (Table 1). The prevalence of HCV was significantly higher among girls (20.3%) than boys (8%) (P = 0.02) (Table 1). However there were no sex differences in the occurrence of HBsAg P = 0.93 (Table 1). The occurrence of HCV was highest (28.5%) in children aged less than two years and in children older than 10 years (Table 1). The same table shows that HBsAg were only seen in two children aged 6–10 years. However the age differences were not statistically significant, with P-values for HBsAg and HCV being 0.72 and 0.69, respectively (Table 1).

**Table 1:** Seroprevalence of HBsAg and HCV infection by age and sex among HIV infected children

**Sex**	**Total sample**	**HBsAg seropositive N (%)**	**HCV seropositive N (%)**
Male	88	1(1.1)	7(8.0)
Female	79	1(1.3)	16(20.3)
		*P = 0.93*	*P = 0.02*
**Age group (yrs)**			
<2	7	0 (0)	2 (28.5)
2–5	37	0 (0)	4 (10.8)
6–10	83	2 (2.4)	10 (12.0)
11–15	34	0 (0)	6 (17.6)
>15	6	0 (0)	1 (16.7)
		*P = 0.72*	*P = 0.69*

**Total**	**167**	**2 (1.2)**	**23 (13.8)**

There were 148 (88.6%) children with history of injection, 26 (15.6%) had blood transfusion, 48 (28.7%) had uvulectomy and 2 (1.2%) were sexually abused. Among those with history of injection 1(0.7%) had HBsAg co-infection and 19(12.8%) had HCV. However, there was no association between these risk factors and the occurrence of hepatitis viral co-infection (Table 2).

**Table 2:** Association between risk factors and HBsAg and HCV co-infection in HIV infected children.

**Risk factor**	**Total sample**	**HBsAg N (%)**	**HCV N (%)**
**Injection**			
Yes	148	1(7)	19(12.8)
No	19	1(5.3)	4(21.1)
		*P = 0.21	*P = 0.30
**Blood transfusion**			
Yes	26	0(0)	5(19.2)
No	141	2(1.4)	18(12.8)
		*P = 1.00	*P = 0.36*
**Uvulectomy**			
Yes	48	1 (2.1)	9 (18.8)
No	119	1 (0.8)	14 (11.8)
		*P = 0.49	*P = 0.32*
**Sexual abuse**			
Yes	2	0 (0)	0 (0)
No	165	2 (1.2)	23 (13.8)
	*P = 1.00		*P = 1.00

Among 167 HIV infected children 59 (35.3%) had no evidence of immunosuppression, 59 (35.3%) had moderate immunosuppression and 49 (29.3%) had severe immunosuppression. There was no statistically significant association between immune status and hepatitis viral co-infection.

None of the investigated children had HBV and HCV dual infection.

Among 167 children, 15 (9%) had elevated ALT. The prevalence of elevated ALT among children co-infected with hepatitis B or C virus was 20.0% as compared with 7.0% among those without viral co-infection. Using logistic regression analysis, children co-infected with hepatitis had 4 times more risk of elevated ALT than those without hepatitis (OR 3.99, 95% CI 1.1–14.2 (Table 3).

**Table 3:** Association between elevated ALT, immune status, ART usage and hepatitis viral co-infection expressed as odds ratios with 95% confidence intervals

		**Univeriate**		**Multivariate**	
	**High ALT N(%)**	**OR**	**95% CI**	**OR**	**95% CI**

**Immunosuppression**					
**No evidence**		1.0	-	1.0	
**Moderate**		0.74	0.16–3.45(P = 0.69)	0.61	0.12–2.96(P = 0.54)
**Severe**		2.68	0.79–9.52(P = 0.13)	2.48	0.68–9.02(P = 0.94)
**ART**					
Not on ART	3(7.1)	1.0	-	1.0	
On ART	12(9.6)	1.38	0.37–5.15(P = 0.09	1.95	0.24–3.77(P = 0.94)
**HBsAg**					
No	14(93.3)	1.0	-		
Yes	1(6.7)	10.79	0.74–8.81(p = 0.14)		
**HCV**					
No	11(73.3)	1.0	-	0.1	
Yes	4(26.7)	2.55	1.02–10.65(P = 0.04)	2.83	0.75–10.62(P0.12)
**Hepatitis B/C viral co-infection**					
No	0(0.0)	1.0		1.0	
Yes	5(20.0)	3.25		3.99	1.12–14.19(P = 0.03)

One hundred and twenty five (74.9%) children were on ART and among them 12(9.6%) had elevated ALT compared with 7.1% among those who were not on ART (Table 3). However, there was no statistically significant association between elevated ALT and being on ART (P = 0.94) (Table 3).

## Discussion

This study showed a high prevalence (15%) of hepatitis co-infection, which is approximately one out seven HIV-infected children. The prevalence of HCV alone observed in this study was 13.8%, which is significantly higher than 7.1%, which was reported by Kitundu *et al *(Jesse Kitundu – Post-transfusion hepatitis C seroprevalence in Tanzanian children. 2001) [7] in post-transfused Tanzanian children. The difference between these studies could be due the nature of the studied populations. In the present study, all children were HIV infected and were therefore more prone to hepatitis viruses, which share modes of transmission. Secondly, the methods used were different, in this study antibodies to HCV was detected using an ELISA technique while in this study agglutination test was used which is relatively less sensitive and less specific due to potential cross reactions [8]. However, it is important that these ELISA based results are confirmed with more sensitive technique such as HCV-RNA by PCR, since it has been shown that some positive ELISA results becomes negative when confirmed by PCR [9].

The seroprevalence of HBsAg alone in this study was 1.2%, which is significantly lower than 12% observed by Kitundu *et al *(Prevalence of Hepatitis B and C among children transfused with anti-HIV negative donor blood at the same hospital [unpublished observation]. A similar study conducted in Kenya by Rana *et al *[10] found a prevalence of hepatitis B to be 4%, among African children infected with HIV.

In this study the prevalence of HCV/HBV was not associated with age (18 months to 17 years). One limitation of this study was that children below 18 months were not investigated due to methodological limitations associated with the use of IgG based ELISA[9]. This limitation may, at least in part, have obscured age trends.

Regarding HCV, females were more affected 17(21.5%) than males 8(9%) a finding that is consistent with a study done by Kitundu *et al *(Prevalence of Hepatitis B and C among children transfused with anti-HIV negative donor blood at MNH hospital in 2000) [unpublished data] in the same setting but the reason for the differences in sex is unclear.

In this study, 148 (88.6%) children had history of injection, 26 (15.6%) had Blood transfusion, 48 (28.7%) had uvulectomy and 2 (1.2%) were sexually abused. However none of these potential risk factors was associated with occurrence of the hepatitis viruses, which in another study conducted in the same hospital by Kitundu *et al *(Prevalence of Hepatitis B and C among children transfused with anti-HIV negative donor blood at MNH hospital [unpublished data]. This finding coupled with the young age of infection, may indicate that possibly these children acquired these viral infections vertically rather than horizontally.

We found no association between HBV and HCV, a finding that is in keeping with observations by Matee *et al *[8] and Wadell *et al *[11] among blood donors in the same hospital. Collectively these studies indicate that the epidemiology of these viruses is different in our setting [8].

The higher prevalence of raised ALT (20.0%) was among children co-infected with hepatitis virus compared with 7.0% without hepatitis co-infection (OR 3.99, 95% CI 1.12–14.19) (Table 3) is expected since hepatitis viruses are known to elevate liver enzyme [12].

We found no association between ART and elevated ALT, which is contrary to the findings of other studies [13-15]. Children recruited in this study were on first line regimen, which does not contain protease inhibitors, which are associated with more risk of elevated ALT than other antiretrovirals [15]. There has also been reports that different genotype of hepatitis C virus differ in their ability to cause hepatotoxicity, with genotype 3 being more hepatotoxic than genotypes 1, 2, and 4. It would be interesting to perform genotyping of the HCV strains to better understand the association with liver pathology.

The high prevalence of hepatitis co-infection among HIV infected children indicates the need to revisit the current guidelines for the clinical management of HIV and AIDS. The regimen (zidovudine, lamivudine, and nevirapine for children < 3 years, with efaviranz replacing nevirapine for children > 3 years and didanosine, abacavir and ritonavir boosted lopinavir as second line) does not take care of the hepatitis co-infection. Although lamivudine works for both HBV and HIV, prolonged lamivudine therapy can result in drug-resistant HBV mutants and has been associated with hepatitis flares [16,17].

## Conclusion

There is high seroprevalence (15%) of hepatitis viral co-infection among HIV infected children attending the Peadiatrics HIV CTC at MNH. Furthermore the current management of paediatrics HIV cases at MNH need to address issues related with hepatitis co-infection.

## Competing interests

The author(s) declare that they have no competing interests.

## Authors' contributions

SPT designed the study and supervised interviews, clinical examination as well as data collection. MIM helped in design of the study and supervised laboratory work. EKM participated in design of the study and supervised clinical work. Finally, all authors participated in preparation of the manuscript read and approved the final manuscript.

## Pre-publication history

The pre-publication history for this paper can be accessed here:


